# Multi-Sensor Integration to Map Odor Distribution for the Detection of Chemical Sources

**DOI:** 10.3390/s16071034

**Published:** 2016-07-04

**Authors:** Xiang Gao, Levent Acar

**Affiliations:** Department of Electrical and Computer Engineering, Missouri University of Science and Technology, 301 West 16th Street, Rolla, MO 65409, USA; acar@mst.edu

**Keywords:** sensor integration, odor source detection, odor distribution map

## Abstract

This paper addresses the problem of mapping odor distribution derived from a chemical source using multi-sensor integration and reasoning system design. Odor localization is the problem of finding the source of an odor or other volatile chemical. Most localization methods require a mobile vehicle to follow an odor plume along its entire path, which is time consuming and may be especially difficult in a cluttered environment. To solve both of the above challenges, this paper proposes a novel algorithm that combines data from odor and anemometer sensors, and combine sensors’ data at different positions. Initially, a multi-sensor integration method, together with the path of airflow was used to map the pattern of odor particle movement. Then, more sensors are introduced at specific regions to determine the probable location of the odor source. Finally, the results of odor source location simulation and a real experiment are presented.

## 1. Introduction

### 1.1. Detection of Odor Source

The detection of airborne chemicals presents a different type of challenge than more traditional detection efforts, such as visual-based detection [1,2] or propagating signal detection [3,4,5]. Chemicals that are airborne tend to drift in various directions due to wind, up-draft, and obstacles. As a result, isolation of the source of such particles becomes considerably difficult and dependent on topography and environment.

There has been some previous research on the detection and modeling of airborne particles, plume location and tracking [6,7,8]. However, most of such research is based on sensor information on moving robots that are guided by the detectors. In [9], the author developed the model using naive physics airflow mapping. In [10], the odor localization used a bi-modal search with the complementary sensing of olfaction and vision. In [11], the author set up a mobile sensing system for localization of an odor source using gas and anemometric sensors. These types of sensing robots are assumed to move about freely following the trail of a chemical signature, while continuously searching for the particles. Both of these assumptions may be invalid in inaccessible and hostile environments with sensors that can either function one time or need long rejuvenation time cycles. To solve these problems, we proposed a novel algorithm of mapping continuous particle paths using discrete sensors for odor source localization, an application of a radial basis function neural network for chemical source detection, and odor source localization using spline interpolation with the complementary Hermite spline function neural network.

In our approach to the problem of chemical particle detection and source location, we use a small number of chemical sensors that are sparsely scattered around an area only known by a two-dimensional map. In real-world problems, we anticipate that an aircraft would drop some of these sensors on the area of interest while taking some aerial pictures. We assume that the sensor data along with the map are transmitted to a nearby location, perhaps to a vehicle that will be traveling through the area of interest. We would like to use the maximum available information content to generate first a model of the chemical particle distribution, and then locate the source of the particles based on the model. Because we obtain the mapping of airflow by utilizing interpolation methods instead of finite element analysis, our approach saves time and computer processing. Finally, through a reasoning system, we localize the area where the chemical source is located.

### 1.2. Odor Sensor and Anemometer Sensor

The important aspects of detecting and tracking chemical sources are odor sensors and anemometer sensors. The odor sensors are for measuring the concentration of chemical particles, and the anemometer sensors are used for the direction of the airflow carrying chemical particles.

Over the last decade, “electronic sensing” or “e-sensing” technologies have undergone important developments from a technical and commercial point of view. The expression “electronic sensing” refers to the capability of reproducing human senses using sensor arrays and pattern recognition systems. Recent research has been conducted to develop technologies, commonly referred to as electronic noses that could detect and recognize odors and flavors [12]. The stages of the recognition process are similar to human olfaction and are performed for identification, comparison, quantification and other applications, including data storage and retrieval. These devices have undergone much development and are now used to fulfill industrial needs. The most commonly available odor sensors detect the presence of airborne substances through changes in the electrical resistances of chemically sensitive carbon-doped polymer films.

An anemometer mounted on the sensor can provide relative velocity between the airflow and the anemometer. Wind speed and wind direction can be measured with a variety of tools. The most common, included with complete home weather stations, is the anemometer, which typically consists of a rotating vane to measure direction and a shaft with cups attached that spins with the wind to measure its speed. An anemometer looks like a weather vane, but instead of measuring which direction the wind is blowing with pointers, it has four cups so that it can more accurately measure wind speed. Each cup is attached to the end of a horizontal arm, each of which is mounted on a central axis, similar to the spokes on a wheel.

## 2. Particle Path Algorithms Using Interpolation and Extrapolation

Using sensors that can collect the sensor’s position, wind velocity, chemical concentration, we can identify the particle paths that describe the pollutant’s propagation in the environment. This particle path map is the first step for detecting the chemical source [13,14,15].

In this paper, we start with the interpolation of two nodes points $\left(x_{0},y_{0}\right)$ and $\left(x_{1},y_{1}\right)$ , where the points are the locations of two sensors with odor particle values of $s_{0}$ and $s_{1}$ , respectively. Since a direct interpolation of a path between the two points would be inconsistent with the odor propagation and the air flow, we generate two more localizations, denoted by $\left(x_{0}^{+},y_{0}^{+}\right)$ and $\left(x_{1}^{+},y_{1}^{+}\right)$ a propagation parameter “*t*” where 0≤t≤1 , and consistent interpolation functions $H_{x}$ and $H_{y}$ , such that

(1) $$\left(x(t),y(t))\approx (H_{x}(t),H_{y}(t)\right)$$

where $x_{0}=H_{x}(0),$ $x_{1}=H_{x}(1),$ $y_{0}=H_{y}(0),$ $y_{1}=H_{y}(1).$

In this approximation, we use Hermite polynomials. In Equation (1), we match the boundary values of the location; however we also need to match the velocities $dx_{0}/dt,dx_{1}/dt,dy_{0}/dt,dy_{1}/dt.$

From the sensor data, we can only collect the derivatives of *y* with respect to *x*, but we need the derivatives of *x* and *y* with respect to *t*. However, these derivatives are easy to determine from using the relationship

(2) ∂y∂x=dydtdxdt

Consequentially, we chose

(3) $$\begin{array}{l} \frac{\partial x}{\partial t}{|}_{t=0}=\frac{dx_{0}}{dt}=\delta x_{0}\\ \frac{\partial y}{\partial t}{|}_{t=0}=\frac{dy_{0}}{dt}=\delta y_{0} \end{array}$$

and,

(4) $$\begin{array}{l} \frac{\partial x}{\partial t}{|}_{t=0}=\frac{dx_{1}}{dt}=\delta x_{1}\\ \frac{\partial y}{\partial t}{|}_{t=0}=\frac{dy_{1}}{dt}=\delta y_{1} \end{array}$$

We, then, proceed to construct the two Hermite polynomials in the usual way, such that

(5) $$\begin{array}{lll} H_{x}(t) & = & ([1-2(t-0)L_{1,0}^{\prime }(0)]L_{1,0}(t{)}^{2})x_{0}+((t-0)L_{1,0}(t{)}^{2})\;\delta x_{0}\\ & & +([1-2(t-1)L_{1,1}^{\prime }(1)]L_{1,1}(t{)}^{2})x_{1}+\;((t-1)L_{1,1}(t{)}^{2})\delta x_{1}\\ & = & ([1-2(t-0)(-1)(\frac{t-1}{0-1}{)}^{2})x_{0}\;+((t-0)\;(\frac{t-1}{0-1}{)}^{2})\;\delta x_{0}\;\\ & & +([1-2(t-1)(1)]\;(\frac{t-0}{1-0}{)}^{2})x_{1}+\;((t-1)(\frac{t-0}{1-0}{)}^{2})\delta x_{1}\\ & = & (1+2t)(t-1{)}^{2}x_{0}+t(t-1{)}^{2}\delta x_{0}+\;(3-2t)t^{2}x_{1}\;+(t-1)t^{2}\delta x_{1} \end{array}$$

where $L_{n,j}$ denotes that the *j*th Lagrange coefficient is the (2n + 1)st order polynomial. 

Similarly, we have

(6) $$H_{y}(t)\;=(1+2t){\left(t-1\right)}^{2}y_{0}+t{\left(t-1\right)}^{2}\delta y_{0}+\;(3-2t)t^{2}y_{1}\;+(t-1)t^{2}\delta y_{1}$$

**Figure 1:**
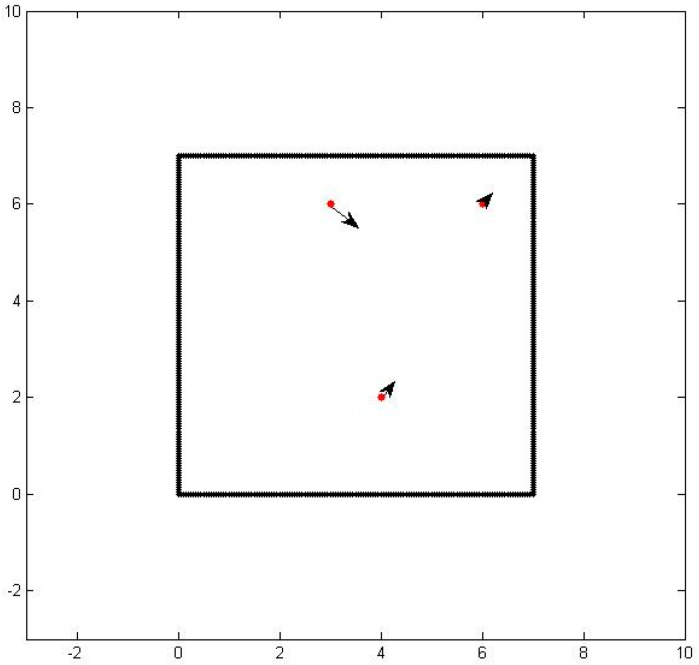
The location of three sensors in a square enclosure.

As a test case, we consider a three sensor configuration system as shown in Figure 1. In the figure, the thick black lines are the boundaries of the area of interest, the red dots are the sensor locations, and the dotted lines designate the particle path lines.

Some chemical sensors are designed to simply detect the existence of a chemical particle and trigger a positive result when the concentration amounts are above a preset threshold level. In our design, instead of the threshold, we make use of the actual concentration levels that are detected. This approach along with some other data enables us to model the flow of the particles and the location of the source. Each sensor provides the co-located sensory information of the wind velocity, the concentration of the particles, and the concentration differential preferably perpendicular to the wind direction. The concentration differential information is obtained not by an additional sensory device but by an off-centered multi-orifice detection hardware configuration. In our derivations, we assume that the differential information is perpendicular to the wind direction, but we can accommodate any non-zero known angular orientation simply by a coordinate transformation. Designating the location of the sensors by (*x*, *y*), we represent the flow of air with (δ_x_, δ_y_). Similarly, we represent the sensed particle concentration with *s* and the concentration gradient with δ_s_.

**Figure 2:**
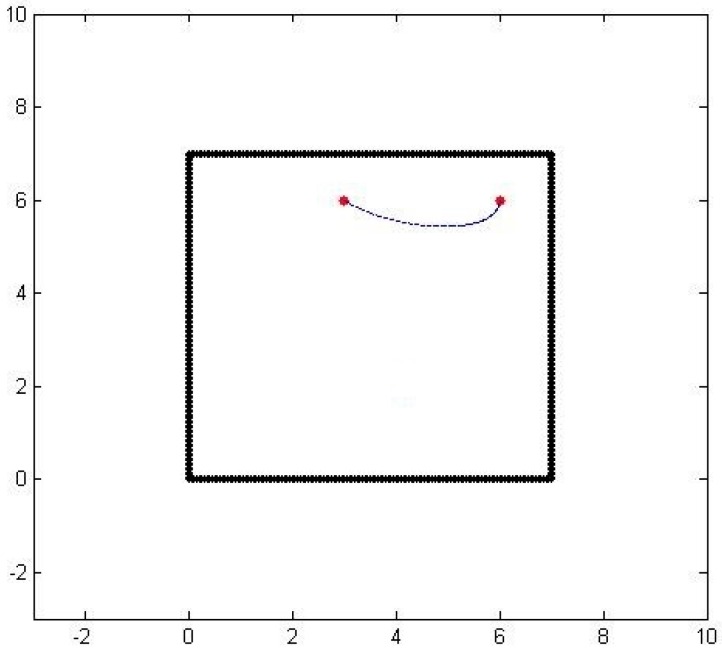
An air-borne particle path with matching terminal velocities.

Once we obtain the sensory information, we start with an approximation of the particle path. In order to avoid multiple solutions, we make a number of assumptions. One assumption is that air-borne particles travel the most direct route. Thus, we configure paths that go through the sensor locations, such that the paths satisfy the locations as well as the differentials. This approach leads to a parametric cubic-polynomial representation of the path in terms of a variable *t*. We use the cubic Hermite splines with the end point differentials weighted three times, such that

(7) $$\begin{array}{l} x(t)=(2(x(0)-x(1))+3(\delta x(0)+\delta x(1)))t^{3}+3(x(1)-x(0))-3(\delta x(1)+2\delta x(0)))t^{2}+3\delta x(0)t+x(0)\\ y(t)=(2(y(0)-y(1))+3(\delta y(0)+\delta y(1)))t^{3}+3(y(1)-y(0))-3(\delta y(1)+2\delta y(0)))t^{2}+3\delta y(0)t+y(0) \end{array}$$

where the parametric curve starts at one sensor location at (*x*(0), *y*(0)) and ends at the other sensor location at (*x*(1), *y*(1)) as t goes from 0 to 1. Figure 2 shows the spline approximation of a particle path from one sensor to another with matching initial and final velocities, but not necessarily matching the sensed values.

## 3. The Framework of Multi-Sensor Integration

### 3.1. The Integration of the Odor Sensor & Anemometer Sensor

Particle-laden flow refers to a class of two phase fluid flow, in which one of phase is continuously connected (referred to as the continuous or carrier phase) and the other phase is made of small, immiscible and typically dilute particles (referred to as the dispersed or particle phase). The problem of detecting an odor source is typically about particle-laden flow. The chemical particle is the dispersed phase, and the air is the carrier phase.

If the mass fraction of the dispersed phase is small, one-way coupling between the two phases is a reasonable assumption; that is, the dynamics of particle phases are affected by the carrier phase, but the reverse is not the case. In our case, the particles are very small and occur in low concentrations; hence the dynamics are governed by the carrier phase. The particle phase is typically treated in a Gaussian distribution [16] along the flow direction

(8) $$C(x,y)=\frac{q}{2\pi kd_{s}}\exp[-\frac{u}{2K}{\left(d_{s}-\Delta d\right)}^{2}]$$

where $d_{s}=\sqrt{{\left(x_{s}-x\right)}^{2}+{\left(y_{s}-y\right)}^{2}}$ , $\Delta d=(x_{s}-x)\cos\theta +(y_{s}-y)\sin\theta$ .

*C* is the concentration (ppm), *q* is the emitted rate (mL/s), *u* is the wind speed (m/s), *K* is turbulent diffusion coefficient (*m^2^/s*), and *θ* is the angle from the x-axis to the upwind direction.

**Figure 3:**
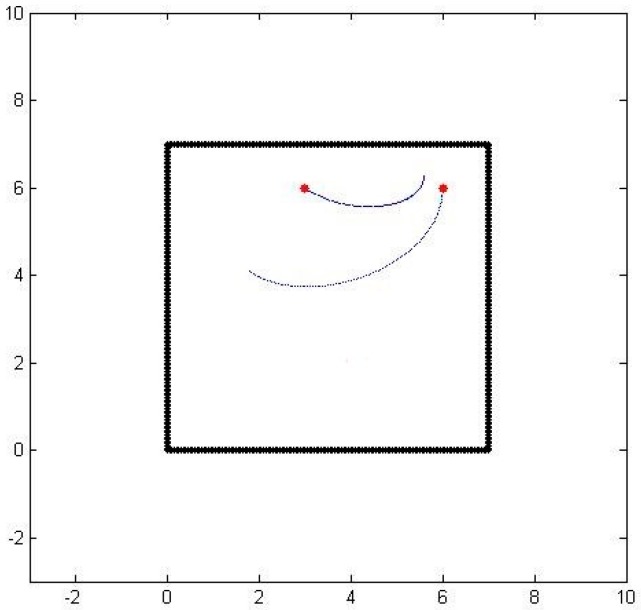
Consistent air-borne particle paths between two sensors.

Even though we now have a path from one sensor to another, there are still several issues to be resolved. The first issue is related to the underlying presumption that a particle would somehow travel from one sensor to the other even though the sensors are at arbitrary locations. To correct this problem, we rely on the dissipation property of the particles. We compute the expected concentration value along the computed path and compare it with the actual sensed concentration value. Based on the error and the measured gradient concentration, we determine a new location perpendicular to the initial path where the expected and sensed concentration values match. We then compute the corrected path going through one of the sensors and the new location. When we repeat the process forwards from one sensor and backwards from another one, we end up getting two consistent paths with correct concentration values. Figure 3 shows the two paths generated by matching the expected and sensed concentration values, as well as the initial and final velocities.

**Figure 4:**
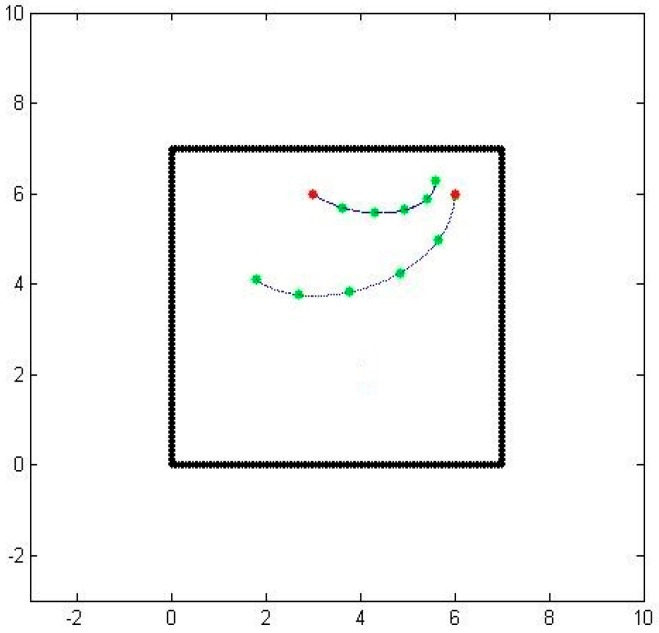
Consistent air-borne particle paths between two sensors.

The second issue is related to the choice of the parameter t. In our parametrization, we chose *t* to start at 0 at one of the sensors and end at 1 at another sensor. We would like to have the parameter be a good representation of actual travel time, since we also would like to obtain connected paths. To correct this problem, we compute the speed at every point along the path as a linear function of the distance from one sensor to the other one while matching the sensed speed values at the two end points. Figure 4 shows the two paths with equally timed distances.

### 3.2. The Integration of the History Sensors & New Sensors

When more sensors are introduced to the region, we need to incorporate the new data and update the particle flow paths. We can integrate the data from the newly added sensors by processing the complete set of sensor data, or we can update the existing air flow paths in the neighborhoods of the new sensors. In this paper, we utilize a novel approach to update the particle paths described by the interpolation functions.

In the original particle path calculations, we generated some primary paths that go through each of the original sensor location and match the sensed values of the particle concentrations. When new sensors are added in between theses primary paths, we need to interpolate and determine secondary paths that go through the new sensors. Since the particle concentration values on these interpolated secondary paths don’t necessary match the observed values from the new sensors, we need to update the primary path data as well.

**Figure 5:**
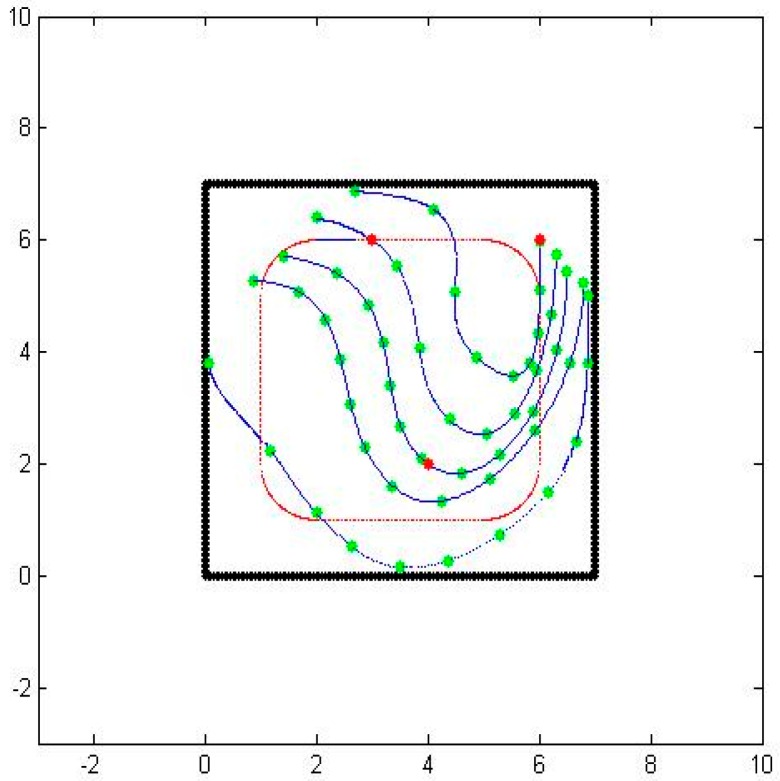
Primary air-borne particle paths going through three sensors and an additional sensor.

As a test case, we initially place 3 sensors and obtain the primary paths from the sensed values. Using the primary paths, we can get secondary paths that map the whole considered area, as shown in Figure 5. We then place another sensor inside the region of interest. Naturally, the sensed values at the new sensor doesn’t match the extrapolated values based on the perpendicular extensions from the primary paths exactly. 

When the new sensor is placed at $\left(x_{*},y_{*}\right)$ , we only need to update the relevant primary paths. The location of the new sensors in relation to the neighboring primary paths determines the paths to be changed. The region where is needed to be changed is defined by the closest primary paths and the perpendicular lines through the sensors on the primary paths. We denote the two sensors on these primary paths as $\left(x_{i},y_{i}\right)$ and $\left(x_{j}y_{j}\right)$ . We model the odor propagation between two primary sensors as two connected particle paths that relate the two primary sensors and the additional sensor. We then use our method to update the parameters of the primary paths to join the piecewise particle paths. 

The updated path equations are modeled by two pieces of particle paths.

The first path, denoted by $L_{1}$ , is from $\left(x_{i},y_{i}\right)$ to $\left(x_{*},y_{*}\right)$ , such that

(9) $$\begin{array}{l} x(t)=(2(x_{i}-x_{*})+3(\delta x_{i}+\delta x_{*}))t^{3}+3(x_{*}-x_{i})-3(\delta x_{*}+2\delta x_{i}))t^{2}+3\delta x_{i}t+x_{i}\\ y(t)=(2(y_{i}-y_{*})+3(\delta y_{i}+\delta y_{*}))t^{3}+3(y_{*}-y_{i})-3(\delta y_{*}+2\delta y_{i}))t^{2}+3\delta y_{i}t+y_{i} \end{array}$$

The piecewise connected second path, denoted by $L_{2}$ , is from $\left(x_{*},y_{*}\right)$ to $\left(x_{j},y_{j}\right)$ , such that

(10) $$\begin{array}{l} x(t)=(2(x_{*}-x_{j})+3(\delta x_{*}+\delta x_{j}))t^{3}+3(x_{j}-x_{*})-3(\delta x_{j}+2\delta x_{*}))t^{2}+3\delta x_{*}t+x_{*}\\ y(t)=(2(y_{*}-y_{j})+3(\delta y_{*}+\delta y_{j}))t^{3}+3(y_{j}-y_{*})-3(\delta y_{j}+2\delta y_{*}))t^{2}+3\delta y_{*}t+y_{*} \end{array}$$

Comparing the particle paths between the updated path and two pieces of particle paths, we define the error term as

(11) E(x,y)=12(∫L1y⋅dxdtdt+∫L2y⋅dxdtdt−∫LΛy⋅dxdtdt)2

where $\overset{\Lambda }{L}$ indicates it’s the path without the additional sensor.

In Equation (11),

(12) ∫LΛy⋅dxdtdt=∫ij((2(yi−y^j)+3(δyi+δy^j))t3+3(y^j−yi)−3(δy^j+2δyi))t2+3δyit+yi)⋅                                                      ((2(xi−x^j)+3(δxi+δx^j))t3+3(x^j−xi)−3(δx^j+2δxi))t2+3δxit+xi)′dt                        

where ( ${\hat{x}}_{j}$ , ${\hat{y}}_{j}$ ) represents the updated location for the jth sensor ( $x_{j}$ , $y_{j}$ ), and the path $\overset{\Lambda }{L}$ is from $\left(x_{i},y_{i}\right)$ to ( ${\hat{x}}_{j}$ , ${\hat{y}}_{j}$ ).

When we substitute Equations (9), (10) and (12) into Equation (11), we get an equation in terms of the unknown parameters ${\hat{x}}_{j}$ , ${\hat{y}}_{j}$ , $\delta {\hat{x}}_{j}$ , and $\delta {\hat{y}}_{j}$ . We assume that the velocity variables $\delta {\hat{x}}_{j}$ and $\delta {\hat{y}}_{j}$ are preserved, and the updated endpoint ( ${\hat{x}}_{j}$ , ${\hat{y}}_{j}$ ) is on the perpendicular line to the primary path going through the jth sensor.

Based on Equation (11), the minimization of the error can be determined by $\underset{{\hat{x}}_{j},\;{\hat{y}}_{j}}{\arg\;\min}E({\hat{x}}_{j},\;{\hat{y}}_{j})$ , the arguments of the minimum is the point at which the error term E attains its smallest value. The most ideal situation is when ∫L1y⋅dxdtdt+∫L2y⋅dxdtdt=∫Ly⋅dxdtdt , where the error becomes zero. The expression of $\underset{{\hat{x}}_{j},\;{\hat{y}}_{j}}{\arg\;\min}E({\hat{x}}_{j},\;{\hat{y}}_{j})$ is

(13) $$\underset{{\hat{x}}_{j},\;{\hat{y}}_{j}}{\arg\;\min}E({\hat{x}}_{j},\;{\hat{y}}_{j})=\;\left\{({\bar{x}}_{j},\;{\bar{y}}_{j})|E({\bar{x}}_{j},\;{\bar{y}}_{j})\leq E({\hat{x}}_{j},\;{\hat{y}}_{j})\right\}$$

We use ( ${\bar{x}}_{j},{\bar{y}}_{j}$ ) to denote the optimal parameters. Since ( ${\bar{x}}_{j},{\bar{y}}_{j}$ ) is on the perpendicular line to the primary path going through the sensor, ( ${\bar{x}}_{j},{\bar{y}}_{j}$ ) satisfies

(14) $${\bar{y}}_{j}=\frac{\delta y_{j}-y_{j}}{\delta x_{j}-x_{j}}({\bar{x}}_{j}-x_{j})+y_{j}$$

Therefore, the updated law is such that

(15) $$\begin{array}{l} {\bar{x}}_{j}=\underset{x_{j},\;y_{j}}{\arg\;\min}E(x_{j},\;y_{j})\\ {\bar{y}}_{j}=\frac{\delta y_{j}-y_{j}}{\delta x_{j}-x_{j}}({\bar{x}}_{j}-x_{j})+y_{j}\\ \delta {\bar{x}}_{j}=\delta x_{j}\\ \delta {\bar{y}}_{j}=\delta y_{j} \end{array}$$

Using ( ${\bar{x}}_{j},{\bar{y}}_{j}$ ) to substitute ( ${\hat{x}}_{j}$ , ${\hat{y}}_{j}$ ), we can get the updated forward particle path as

(16) $$\begin{array}{l} x(t)=(2(x_{i}-{\bar{x}}_{j})+3(\delta x_{i}+\delta {\bar{x}}_{j}))t^{3}+(3({\bar{x}}_{j}-x_{i})-3(\delta {\bar{x}}_{j}+2\delta x_{i}))t^{2}+3\delta x_{i}t+x_{i}\\ y(t)=(2(y_{i}-{\bar{y}}_{j})+3(\delta y_{i}+\delta {\bar{y}}_{j}))t^{3}+(3({\bar{y}}_{j}-y_{i})-3(\delta {\bar{y}}_{j}+2\delta y_{i}))t^{2}+3\delta y_{i}t+y_{i} \end{array}$$

**Figure 6:**
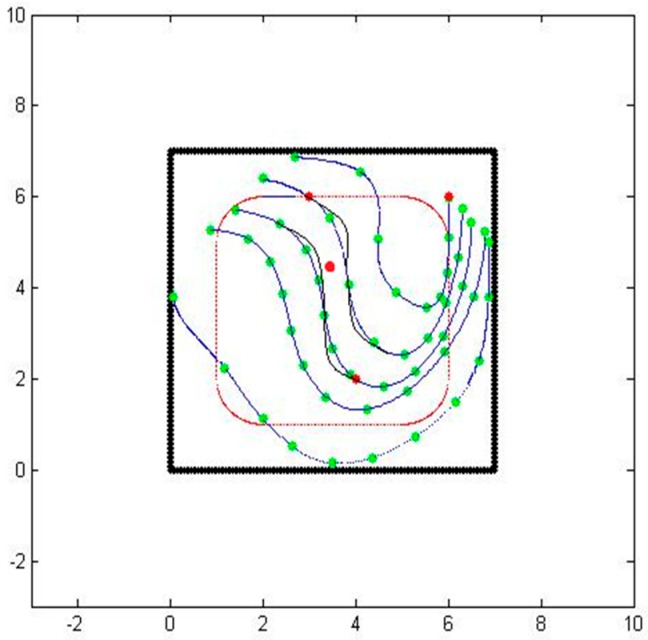
Updated air-borne particle paths going through three sensors and an additional sensor.

Similarly, we can get the backward particle paths using the same method to update the variables for the *i*th sensor. Figure 6 shows the updated map of the particle paths. 

## 4. Compare and Validate Our Approach Using Computational Fluid Dynamics

To compare and validate our approach, we use exact analytical methods for simpler cases and use finite-element method based business software (such as COMSOL) for more complicated cases.

The analysis of airborne particle motion is identical to fluid motion analysis in physics. The fluid motion is governed by the Navier–Stokes nonlinear partial differential Equations (17) and (18), such that motion in the two dimensional space satisfies

(17) $$\begin{array}{l} \frac{\partial u}{\partial x}+\frac{\partial v}{\partial y}=0\\ u\frac{\partial u}{\partial x}+v\frac{\partial u}{\partial y}=-\frac{1}{\rho }\frac{\partial P}{\partial x}+\nu (\frac{\partial^{2}u}{\partial x^{2}}+\frac{\partial^{2}u}{\partial y^{2}})\\ u\frac{\partial v}{\partial x}+v\frac{\partial v}{\partial y}=-\frac{1}{\rho }\frac{\partial P}{\partial y}+\nu (\frac{\partial^{2}v}{\partial x^{2}}+\frac{\partial^{2}v}{\partial y^{2}}) \end{array}$$

where *u* and *v* are the components of the velocity in the *x* and *y* directions, *ρ* is the fluid density, and *P* is the pressure.The analytical solutions to the Navier-Stokes equations depend on the initial and the boundary conditions, and exact solutions exist only for simple cases.

In this paper, we assume that the particle flow dynamic is two dimensional, and is uncompressible, inviscid, and irrotational. If *D* is a simply connected domain in 2 demensions and the flow is irrotational, the integral $\int_{\left(a,b\right)}^{\left(x,y\right)}ud\xi +vd\zeta$ is independent of the path in D. If we integrate from a fixed point (*a*, *b*) to a variable point (x, y), then the integral becomes a functions of the point (x, y).

(18) $$\Phi (x,y)=\int_{\left(a,b\right)}^{\left(x,y\right)}\left(ud\xi +vd\zeta \right)$$

We define the function Φ(x,y) as velocity potential of the motion. Since the integral is independent of the path, and udx+vdy is an exact differential, the differential of function Φ(x,y) satisfies

(19) $$udx+vdy=\frac{\partial \Phi }{\partial x}dx+\frac{\partial \Phi }{\partial y}dy$$

From Equation (19), we get

(20) $$u=\frac{\partial \Phi }{\partial x},\;v=\frac{\partial \Phi }{\partial y}$$

By substituting *u* and *v* in Equation (20) into Equation (17), we observe that Φ(x,y) satisfies the Laplace’s equation

In other words, we can use the Laplace’s equation to model fluid motion.

(21) $$\nabla^{2}\Phi =\frac{\partial^{2}\Phi }{\partial x^{2}}+\frac{\partial^{2}\Phi }{\partial y^{2}}=0$$

Let Ψ(x,y) be a conjugate function of Φ(x,y) . The function Ψ(x,y) is called the stream function of the flow. The curves where Ψ(x,y) is constant are called the streamlines of the fluid. We know that both Ψ(x,y) and Φ(x,y) have continuous second derivatives as shown in [13]. Consequentially the complex function

(22) F(x,y)=Φ(x,y)+iΨ(x,y)

is analytic in the region of the flow. This function is called the complex potential of the flow.

We can determine the velocity of the flow by differentiating Equation (22) and using the Cauchy-Riemann Equation (19); such that

(23) $$F_{x}(x,y)=\frac{\partial \Phi }{\partial x}+i\frac{\partial \Psi }{\partial x}=\frac{\partial \Phi }{\partial x}-i\frac{\partial \Phi }{\partial y}=u-iv$$

A general solution to Equation (23) can be complicated and unnecessary for the simple cases that we are considering. Indeed, we can assume the form of the function *F* from the initial flow and determine specifics by substituting the functions into the differential equations. Because of the uniqueness of the solution under initial and boundary conditions, if the function *F* satisfies Equation (21) and the boundary conditions, then it is the unique solution.

Case 1. Fluid flow with free boundary conditions

**Figure 7:**
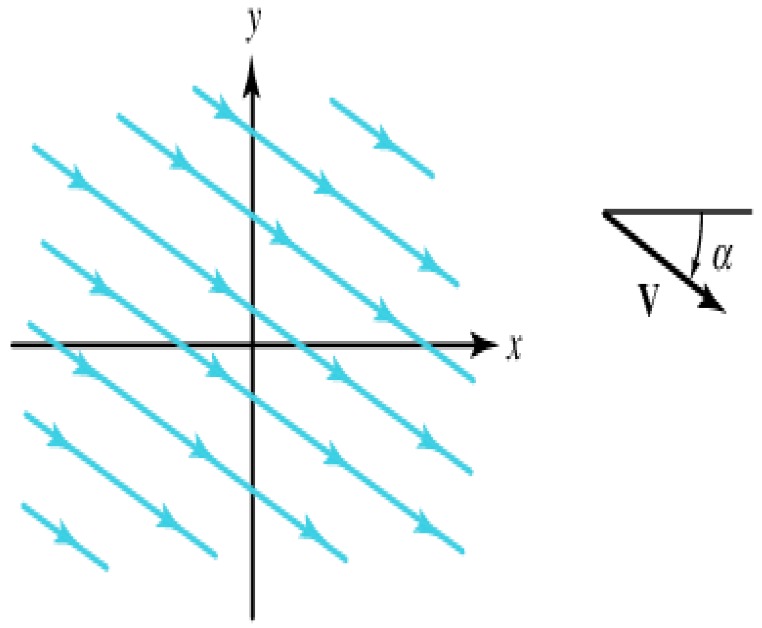
A uniform parallel flow.

As the first case, we choose the no boundary case with a uniform infinite width flow of particles at a certain angle α, as shown in Figure 7. By choosing $F(x,y)=k_{1}x+ik_{2}y$ , where $k_{1},k_{2}$ are real numbers, we describe a uniform flow in the x−y plane.

If the flow is irrotational, then the equation ∇×F=0 is automatically satisfied by F=−∇ϕ , where *φ* is the velocity potential, hence $F_{y}=-\frac{\partial \psi }{\partial y}$ and $F_{y}=-\frac{\partial \phi }{\partial y}$ . On the other hand, if the flow is incompressible then $F_{x}=-\frac{\partial \psi }{\partial y}$ and $F_{y}=\frac{\partial \psi }{\partial x}$ , where *ψ* is the stream function.

Consequentially, by substituting $F(x,y)=k_{1}x+ik_{2}y$ into the differential equations, we can determine that the velocity potential is $\Phi (x,y)=k_{1}x-k_{2}y$ and the stream function is $\psi (x,y)=k_{2}x+k_{1}y$ , such that the streamlines and the potential lines are orthogonal. The velocity of the flow can be obtained from $V(x,y)={\left(F^{\prime }(x,y)\right)}^{*}={\left(k_{1}x+ik_{2}y\right)}^{*}=k_{1}x-ik_{2}y,$ where ${\left(\cdot \right)}^{*}$ is the complex conjugate. 

The streamlines are the parallel lines described by the equation $k_{2}x+k_{1}y=\text{constant}$ and are inclined at an angle $\alpha =-\arctan(k_{2}/k_{1})$ .

For two arbitrary locations of sensors in the x−y plane $\left(x_{1},y_{1}\right)$ and, we use our method to determine the fluid propagation path between the two points. However, to satisfy the sensed chemical concentration values, we need to modify the paths. In this case, when the two locations are on the same streamline, the streamlines become $k_{2}x+k_{1}y=\alpha \tan(t)$ , where α is a constant. In this case, we get zero error between the two methods, because the two methods give the same result. 

**Figure 8:**
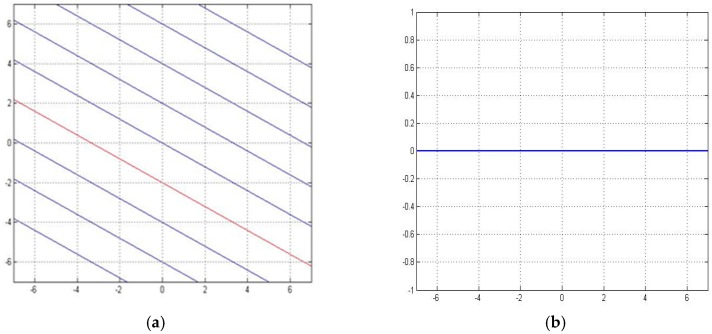
The performance of interpolation and extrapolation for a uniform parallel flow. (**a**) Particle paths obtained by our method, (**b**) The error curve.

Figure 8a shows the map of particle paths obtained by our method, Figure 8b shows the error between our method and the computational fluid dynamics.

Case 2. Fluid flow with infinite wall

**Figure 9:**
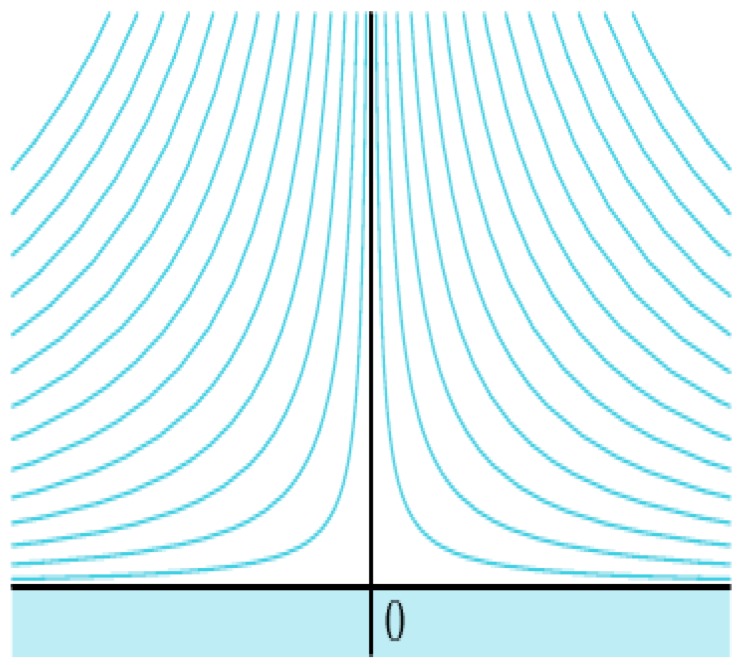
The fluid flow around an infinite wall.

In this case, the complex potential function is $F(x,y)=\frac{A}{2}(x^{2}-y^{2})+iAxy$ , as shown in Figure 9, where *A* is a positive real number. The velocity potential and the stream functions are given by $\Phi (x,y)=\frac{A}{2}(x^{2}-y^{2})$ and ψ(x,y)=Axy .

The streamlines, where ψ(x,y) is constant, are from a family of hyperbolic functions with asymptotes along the coordinate axes. The velocity vector $V(x,y)={\left(F^{\prime }(x,y)\right)}^{*}=A(k_{1}x-k_{2}y)$ indicates that in the upper half-plane, the fluid flows down along the streamlines and spreads out along the x axis, as against the wall.

Similarly, we choose two arbitrary points in the x−y plane $\left(x_{1},y_{1}\right)$ and $\left(x_{2},y_{2}\right)$ as the two sensor’s locations, and use the interpolation method to get the propagation path between the two points. However, to be consistent with the chemical concentrations, we need to modify the endpoints of the path. When we choose the two points on a same streamline, the streamline can be described as a cubic function $y=ax^{3}+bx^{2}+cx+d$ . The error term becomes $\int_{\left(x_{1},y_{1}\right)}^{\left(x_{2},y_{2}\right)}(k/x-ax^{3}-bx^{2}-cx-d{)}^{2}dx$ , where the *K* is a real number and (*a*, *b*, *c*, *d*) are four parameters related with the coordinates of the two points and their derivatives. In this case, substituting the (*a*, *b*, *c*, *d*), we get the error term between our method and the computational fluid dynamics is $k^{2}(\frac{1}{5}(x_{2}^{5}-x_{1}^{5})-\frac{\left(x_{1}+x_{2}\right)}{2}(x_{2}^{4}-x_{1}^{4})+\frac{1}{3}(x_{1}^{2}+x_{2}^{2}+4x_{1}x_{2})(x_{2}^{3}-x_{1}^{3})-x_{1}x_{2}(x_{1}+x_{2})(x_{2}^{2}-x_{1}^{2})+x_{1}^{2}x_{2}^{2}(x_{2}-x_{1}).$

**Figure 10:**
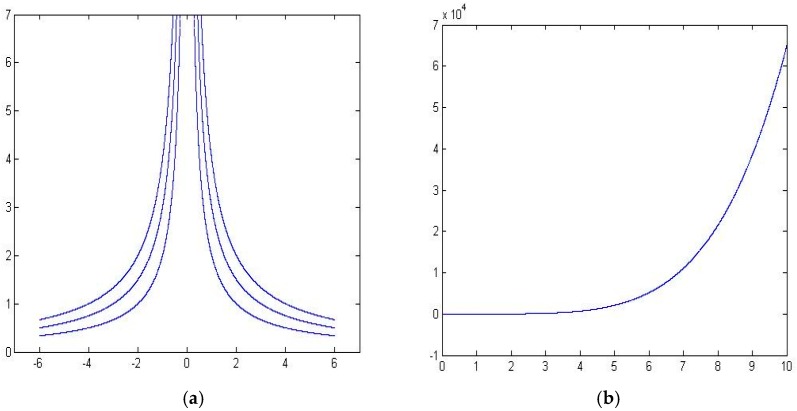
The performance of interpolation and extrapolation for the fluid flow around an infinite wall. (**a**) Particle paths obtained by our method, (**b**) The error curve.

Figure 10 shows the error curve between the fluid dynamic method and our proposed method. Figure 10a shows the particle path map by using our method, and Figure 10b shows the error. From the figures, we can conclude that the error increases as the distance between two sensors increases.

Case 3. Inviscid flow past a cylindrical obstacle

**Figure 11:**
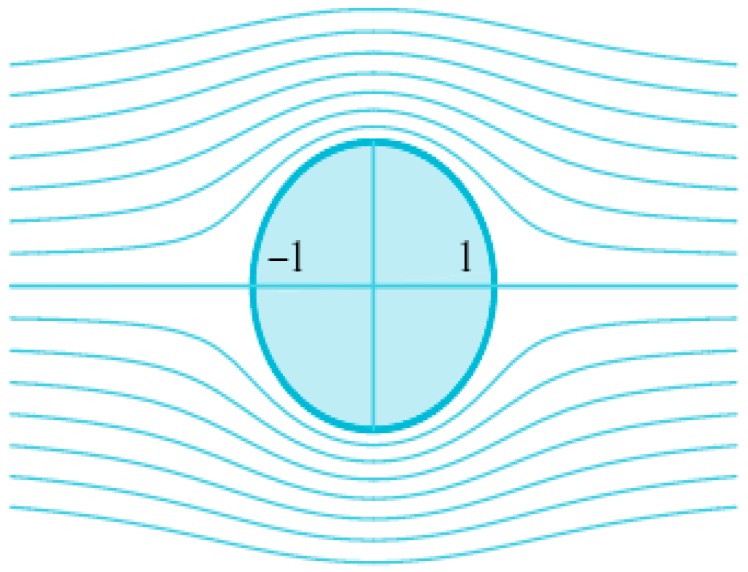
Fluid flow around a circular obstacle.

In this case, we consider a circular obstacle in the direction of the flow, as shown in Figure 11. We can use polar coordinates to express the complex potential function *F*(*z*) as

$$\begin{array}{l} F(re^{i\theta })=A(re^{i\theta }+\frac{1}{re^{i\theta }})\\ \;=A(rcos\theta +irsin\theta +\frac{1}{rcos\theta +irsin\theta })\\ \;=A(rcos\theta +irsin\theta +\frac{r(\cos\theta -ir\sin\theta )}{r^{2}})\\ \;=A(r+\frac{1}{r})\cos\theta +iA(r-\frac{1}{r})\sin\theta \end{array}$$

The streamline is $\psi_{\rho }(r,\theta )=A(r-\frac{1}{r})\sin\theta =\mathrm{constant}$ , where A is a positive real number. When the constant is zero, it consists of the paths r>1,   θ=0   and  r>1,  θ=π along the x axis and the curve $r-\frac{1}{r}=0$ , which is the unit circle |z|=r=1 . In the other words, the unit circle can be considered a boundary curve for the particle flow. The approximation is valid for large values of r, so we can approximate the flow with a uniform horizontal flow having speed |V(x,y)|=A at points that are distant from the origin.

**Figure 12:**
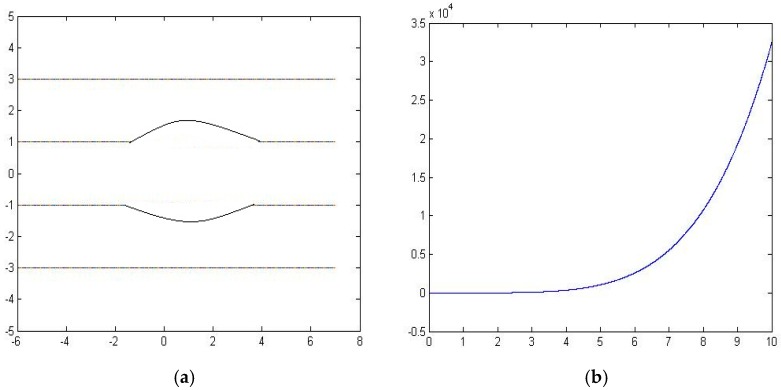
The performance of interpolation and extrapolation for the fluid flow around a circular obstacle. (**a**) Particle paths obtained by our method, (**b**) The error curve.

Similarly, we chose two arbitrary points in the x−y plane ( $\left(x_{1},y_{1}\right)$ and $\left(x_{2},y_{2}\right)$ ) as the two sensor’s locations, and we use the interpolation method to get the particle propagation path between the two points. Figure 10a shows the fluid path map by using our method. And Figure 12b shows the error between the fluid dynamic method and our method.

From the error analysis, we observe that the error can be considerably large when the sensors are placed wide apart. In the other words, the more sensors we use, the better result are expected to obtain. Further analyzing the three examples, we conclude that when the Hermite polynomials can interpolate the stream function perfectly, we can get zero error as in the first example. On the other hand, when the streamline functions cannot be interpolated by using the 3rd order polynomials, we get the larger errors as in the third example.

## 5. Examples and Numerical Results

**Figure 13:**
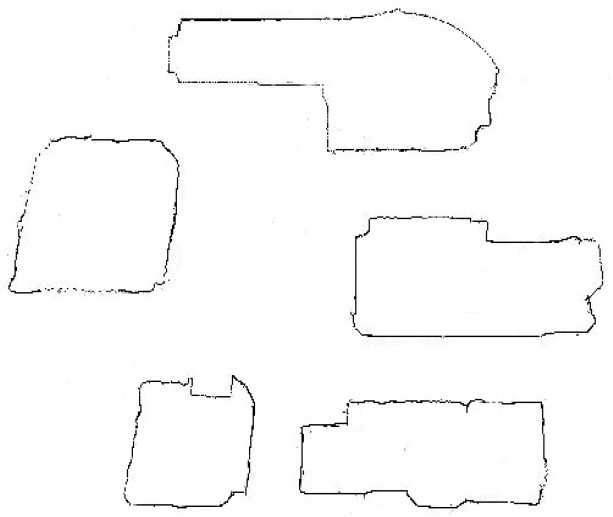
A portion of the Missouri University of Science and Technology campus after an edge detection algorithm.

In this section, we apply our method to a real world region. We choose a section of the real map of Missouri University of Science and Technology (Missouri S&T) campus, and use an edge detection technology to generate a boundary and open area map as shown in Figure 13.

In the experiment, we first scatter ten sensors in the considered area, then using our method, we determine the primary particle paths based on the data from the early two sensors. When we incorporate the other sensors’ information, we modify the particle paths. Finally, we use the COMSOL program to simulation the chemical particle’s propagation under the same conditions. 

**Figure 14:**
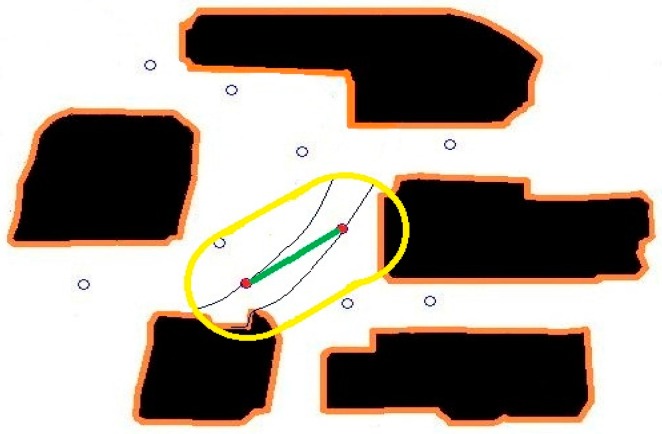
The map of particle paths using two sensors.

**Figure 15:**
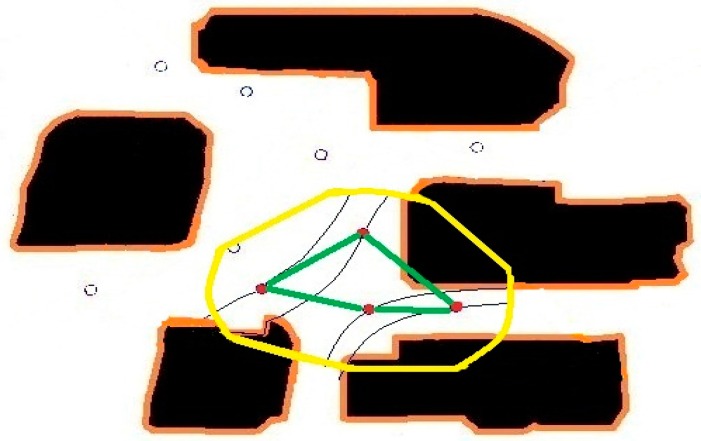
The map of particle paths using four sensors.

**Figure 16:**
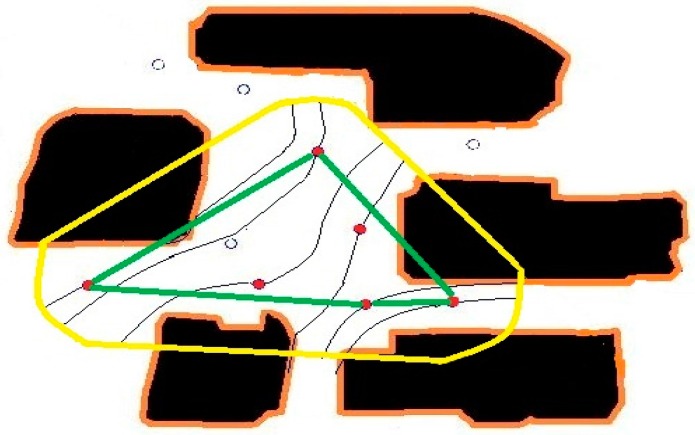
The map of particle paths using six sensors.

**Figure 17:**
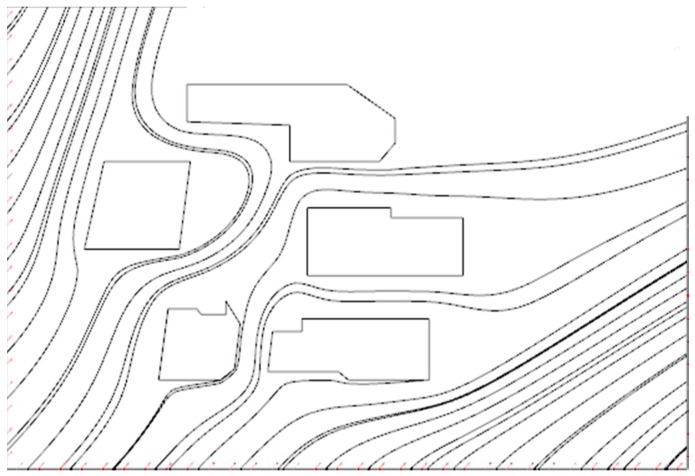
Air-borne particle paths going through ten sensors in a real map processed by COMSOL.

In Figure 14, we use only two sensors within the considered area to map the particle paths. In Figure 15 and Figure 16, we include two and four more sensors in the same area, and update the parameters. Figure 17 shows that the more sensor we use, the better our approximation gets closer to the real particle paths.

## 6. Conclusions

There are many useful and humanitarian purposes in locating the source of a chemical source. Currently, the majority of work in this area uses reactive control schemes that track an odor plume along its entire length, which is slow and difficult in cluttered environments. This paper presents a high-level control scheme that uses an interpolation and extrapolation method to model the particle path. It also presents a reasoning system that uses path modeling to obtain the velocity and chemical concentrations and to predict the most probable locations of the odor source. This approach has been shown to be effective for odor localization in a known environment, without the need for a robot to travel to the source.

With further development there is great potential for this approach to lead to many valuable applications by generalization to a wider range of environmental configurations. The paper presents developments to solve the problem of obstacles and pathways in the environment In addition, this paper is the first example of using interpolation and extrapolation methods to model the particle path applied in a real environment.

The results of simulating particle paths in the real map of campus approximate the results from the fluid dynamic analysis software, COMSOL.
